# Epidemiology of 45,616 suspect cases of Hand, Foot and Mouth Disease in Chongqing, China, 2011–2015

**DOI:** 10.1038/srep45630

**Published:** 2017-04-19

**Authors:** Jian Tao, Xiao-yan He, Yu Shi, Guohun Zhu, Shan Liu, Zhenzhen Zhang, Shi Tang, Rong Zhang, Bin Peng, Zhidai Liu, Junjie Tan, Qian Chen, Xingbin Wang, Liming Bao, Lin Zou, Penghui Zhang

**Affiliations:** 1 Center for Clinical Molecular Medicine, Children’s Hospital, Chongqing Medical University, Ministry of Education Key Laboratory of Child Development and Disorders, Key Laboratory of Pediatrics in Chongqing, Chongqing International Science and Technology Cooperation Center for Child Development and Disorders, Chongqing, 400014, China; 2 Laboratory Medicine Center, Shenzhen People’s Hospital, Shenzhen, 518020, China; 3 Laboratory Medicine Center, Children’s Hospital, Chongqing Medical University, Chongqing, 400014, China; 4 School of Information Technology and Electrical Engineering, The University of Queensland, QLD 4067, Australia; 5 Department of Infectious diseases, Children’s Hospital, Chongqing Medical University, Chongqing, 400014, China; 6 Department of Health Statistics, School of Public Health, Chongqing Medical University, Chongqing, 400016, China; 7 Department of Pathology and Laboratory Medicine, Geisel School of Medicine Dartmouth College, One Medical Center Drive, Lebanon, NH 03756, USA

## Abstract

Epidemiology and etiology of hand, foot, and mouth disease (HFMD) based on large sample size or evaluation of detection for more enterovirus serotypes are not well investigated in Chongqing of China. 45,616 suspect HFMD patients were prospectively enrolled among whom 21,615 were laboratory confirmed HFMD cases over a 5-year period (January 2011 to December 2015). Their epidemiological, clinical, and laboratory data were extracted and stratified by month, age, sex, disease severity, and enterovirus serotype. Subsequently 292 non-EV-A71/CV-A16 HFMD confirmed cases were randomly selected in three consecutive outbreaks to detect CV-A6 and CV-A10, using RT-PCR. Results showed that the HFMD epidemic peaked in early summer and autumn. The median age of onset was 2.45 years with a male-to-female ratio of 1.54:1, and with children under 5 years of age accounting for 92.54% of all confirmed cases. EV-A71 and CV-A16 infection accounted for only 36.05% (7793/21615) of total confirmed cases while EV-A71 accounted for 59.64% (232/389) of severe cases. Importantly, the proportion of EV-A71 infection generally increased with age which showed rapid growth in severe cases. CV-A6 and CV-A10 were tested positive in Chongqing, but CV-A6 had greater positive rates of 62.33% while CV-A10 had 4.79% in non-EV-A71/CV-A16 HFMD confirmed cases.

Hand, foot, and mouth disease (HFMD) is a common childhood infectious disease mainly caused by various human enteroviruses (EVs). EVs spread quickly through fecal-oral transmission but mostly cause HFMD in children under 5 years of age^1^. After EVs enter the intestine and break through the gut barrier to enter the blood, they are able to reach the spinal cord, brain, meninges, heart, liver, skin, nail and other organs thus causing relevant clinical symptoms. Most patients show self-limiting illness, typically fever, skin eruptions on palms, soles or buttocks, and vesicles in the mouth^2^. However, some patients rapidly develop neurological and systemic complications that can be fatal, such as acute flaccid paralysis, encephalitis, myocarditis, and encephalomyelitis^3^. Till so far, there is no specific treatment for HFMD, nor effective prevention of severe cases. Even HFMD vaccine designed for EV-A71 is commercially available in China since 2015^4^, no reports on implementation of it or the reduction of HFMD cases due to it. Understanding its epidemiology and etiology is essential for controlling morbidity rates of HFMD.

EVs are members of the genus *Enterovirus* within the family *Picornaviridae*, order *Picornavirales*, which consists of 4 species: *EV-A, EV-B, EV-C*, and *EV-D*^5^. To date, EVs comprise more than 100 serotypes, and prevalent pathogens mainly distribute in *EV-A* and EV-B. Enterovirus A71 (EV-A71) and coxsackievirus A16 (CV-A16) of *EV-A* are widely considered to be typically the most common agents causing HFMD, and EV-A71 is the dominant causative pathogen in severe cases^1^. However, coxsackievirus A10 (CV-A10)^6^,^7^ and coxsackievirus A6 (CV-A6)^8^,^9^,^10^,^11^,^12^,^13^,^14^,^15^ of *EV-A* have been increasingly reported worldwide in the past few years, which indicate that prevalent pathogens are diverse and shifting. Meanwhile, several atypical cutaneous manifestations of HFMD associated with CV-A6^16^,^17^,^18^,^19^,^20^ have been described which has highlighted the necessity of virological surveillances to simultaneously detect additional EVs serotypes besides EV-A71 and CV-A16. However, there were no reports on the evaluation of simultaneous detection of CV-A6 and CV-A10 in the clinical laboratory center while only EV-A71, CV-A16 and EVs universal (EVs) were detected conventionally.

The epidemiological character of HFMD in Chongqing was firstly described in 3,472 inpatients^21^. However, during the period 2011–2015, 21,615 out of 45,616 HFMD suspect patients admitted to The Children’s Hospital of Chongqing Medical University, who underwent laboratory etiological detection, were diagnosed as confirmed cases. The epidemiology and etiology of this large HFMD population remains unknown. Moreover, most patients with obvious HFMD clinical manifestations who contracted EVs tested positive and could not obtain causative EVs serotype identified in a clinical laboratory center.

In this study, 45,616 HFMD suspect cases received laboratory etiological detection and 21,615 confirmed HFMD patients were enrolled to characterize the epidemiology and etiology of HFMD, focusing on seasonal, age, gender and especially serotype patterns. Valuation of CV-A6 and CV-A10 identification was also investigated in other EVs positive cases with randomly collected samples in consecutive outbreaks from November 2015. This study evaluates the epidemic of HFMD based on large sample size and simultaneous detection for CV-A6 and CV-A10, which will help evaluate the effective protection of the EV-A71 vaccine against HFMD and the necessity of virological detection of more EVs serotypes other than EV-A71 and CV-A16 in clinical laboratories.

## Materials and Methods

### Case definitions

The diagnosis of HFMD was carried out and guided according to the criteria issued by the National Health and Family Planning Commission of the People’s Republic of China. Suspect HFMD cases were defined as patients with maculopapular or vesicular rash on the hand, foot, mouth, and buttocks with or without fever, while confirmed cases were defined as suspect case with laboratory confirmation of EVs infection (including EV-A71, CV-A16, or EVs) completed by RT-PCR assays. The confirmed cases with neurological complications (aseptic meningitis, encephalitis, encephalomyelitis, acute flaccid paralysis, or dysfunction of the autonomic nervous system), and cardiopulmonary complications (pulmonary edema, pulmonary hemorrhage, or cardiorespiratory failure, or both, were characterized as severe cases of HFDM, otherwise, patients were categorized as mild cases.

### Sample collection and EVs detection

Depending on the symptoms and clinical status of suspect HFMD patients, we collected the appropriate clinical specimens, including throat swab, rectal swab, fecal sample, vesicular fluid, or cerebrospinal fluid, and screened for EVs. RNA were extracted from the supernatants of the swab or stool specimens soaked in normal saline (NS) and dissolved in 30 μl diethypyrocarbonate (DEPC) water. One-step RT-PCR assays were performed to detect EVs RNAs, using the EV-A71, CV-A16 and EVs test commercial kits (Sansure Biotech, China). Product performance indicators of diagnostic kit for EV-A71 (Registration number: 20133400621), CV-A16 (Registration number: 20133400619), and EVs (Registration number: 20133400616) are the same as follows: Negative and positive coincidence rates are 100%, repeatability of intra and inter batch assays are good, variable coefficient of Ct value are all less than 10%, minimum detectable amount are 2.00E + 02 copies/ml, and there is no cross reaction between other viruses with similar infection symptoms.

### Clinical data collection

The Children’s Hospital of Chongqing Medical University is the designated center for HFMD diagnosis and treatment in Chongqing. HFMD suspect patients received laboratory etiological detection to identify laboratory confirmed cases between Jan 1, 2011, and Dec 31, 2015. The clinical and laboratory data was extracted from medical records, including basic demographic information (sex and date of birth), date of illness onset, duration of illness, symptoms, case classification (suspect or confirmed), severity (mild or severe), death status, date of symptoms onset, date of diagnosis, and date of death (if applicable), sample type (throat swab, rectal swab, fecal sample, vesicular fluid, or cerebrospinal fluid), and virus serotype (EV-A71, CV-A16 or EVs) for confirmed cases.

### Non-EV-A71/CV-A16 sample collection and CV-A6/CV-A10 detection

On the day the laboratory test reports were issued, HFMD patients who tested EV-A71 and CV-A16 negative but EVs positive, were sorted out immediately. Their clinical specimens and RNA samples extracted for virological surveillances were subsequently collected and stored at −80 °C during the three HFMD outbreaks in November 2015, June 2016 and October 2016. A total of 292 eligible swab samples were randomly collected in these outbreaks for RNA extraction to avoid RNA samples for virological detection from degrading. Meanwhile, their RNA samples were collected for CV-A6/CV-A10 detection. One-step RT-PCRs were performed to detect CV-A6 and CV-A10, using the test commercial kits (Sansure Biotech, China). Product performance indicators of diagnostic kit for CV-A6 and CV-A10 were the same as those of EV-A71, CV-A16 and EVs.

### Data Analysis

All the confirmed cases of the onset of HFMD illness from Jan 1, 2011, to Dec 31, 2015 were enrolled in the analysis. We estimated the number of month-specific, age-specific, gender-specific and serotype-specific cases of HFMD by applying distribution in samples positive for the EVs in the corresponding year, respectively. To quantify seasonal patterns of HFMD by the month, we applied serotype-specific cases distribution in each month of the year. To quantify age patterns of HFMD, we applied age-specific cases distribution in each age group. To estimate proportions of each serotype pathogen in mild and severe HFMD cases according to year and age group, we applied serotype-specific cases in proportion to the total positive cases of the corresponding group. In CV-A6 and CV-A10 analysis, we estimated their positive rates in three consecutive HFMD outbreaks in November 2015, June 2016 and October 2016, respectively.

### Ethics Statement

The study protocol was approved by the Institutional Review Board of the Children’s Hospital of Chongqing Medical University. The methods were carried out in accordance with the approved guidelines. Since this study was a retrospective analysis and all information were kept anonymous to protect patient confidentiality, written consents from the patients were waived.

## Results

**Figure 1:**
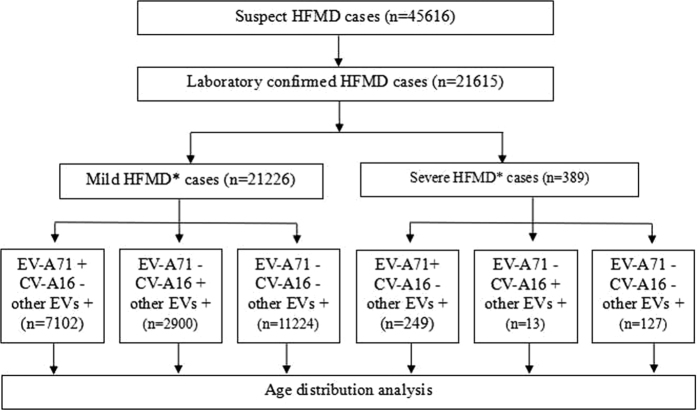
Flow diagram of HFMD patients in whom epidemiology and etiology were investigated. EV-A71 = enterovirus A71. CV-A16 = coxsackievirus A16. Other EVs = other enterovirus universal. *Cliassification according to the criteria issued by National Health and Family Planning Commission of the People’s Republic of China.

**Figure 2:**
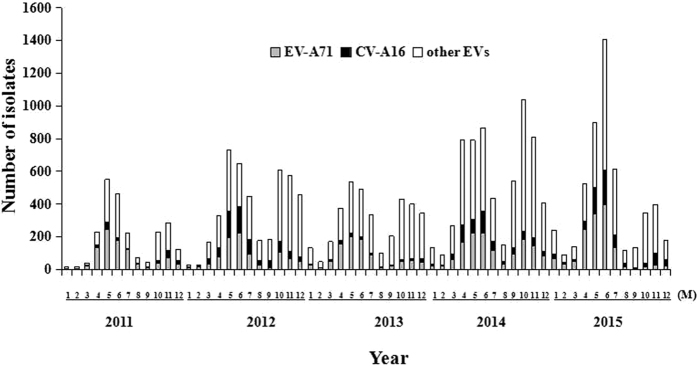
Monthly distribution and constituent ratio of enterovirus serotype associated with HFMD confirmed cases in Chongqing of China, 2011–2015.

**Figure 3:**
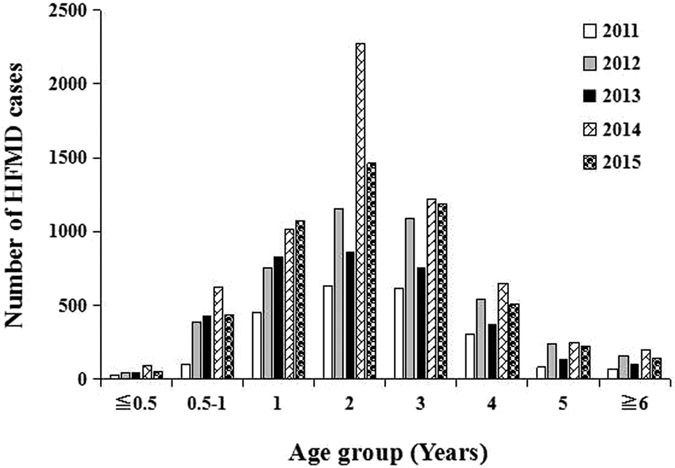
Age distribution in HFMD confirmed cases in Chongqing of China, 2011–2015.

Total of 45,616 suspect HFMD cases were admitted in the Children’s Hospital of Chongqing Medical University during 2011–2015, of which 21,615 (47.38%) were laboratory confirmed cases and 389 (1.80%) were severe cases (Fig. 1). EV-A71, CV-A16 and other EVs co-circulated throughout the study period, and all of them had two outbreaks peaking in Spring and early Summer followed by a smaller peak in Autumn of each year (Fig. 2). The incidence of HFMD varied greatly with age, with the highest rates in children aged 0.5 to 4 years. The median age of reported cases was 2.45 years (range 1 month to 17 years). Most HFMD cases were in children younger than 5 years, accounting for 92.54% of all confirmed cases, and the incidence was very low in infants younger than 0.5 years and patients aged more than 5 years. Moreover, the one-year and three-year group accounted for 19.09% and 22.56% respectively, followed by the two-year age group which accounted for the largest proportion of 29.56% (Fig. 3). The incidence of HFMD was higher in boys than that in girls throughout the five-year period with the male-to-female ratio (1.54:1) of all confirmed cases included in the study (Table 1).

**Figure 4:**
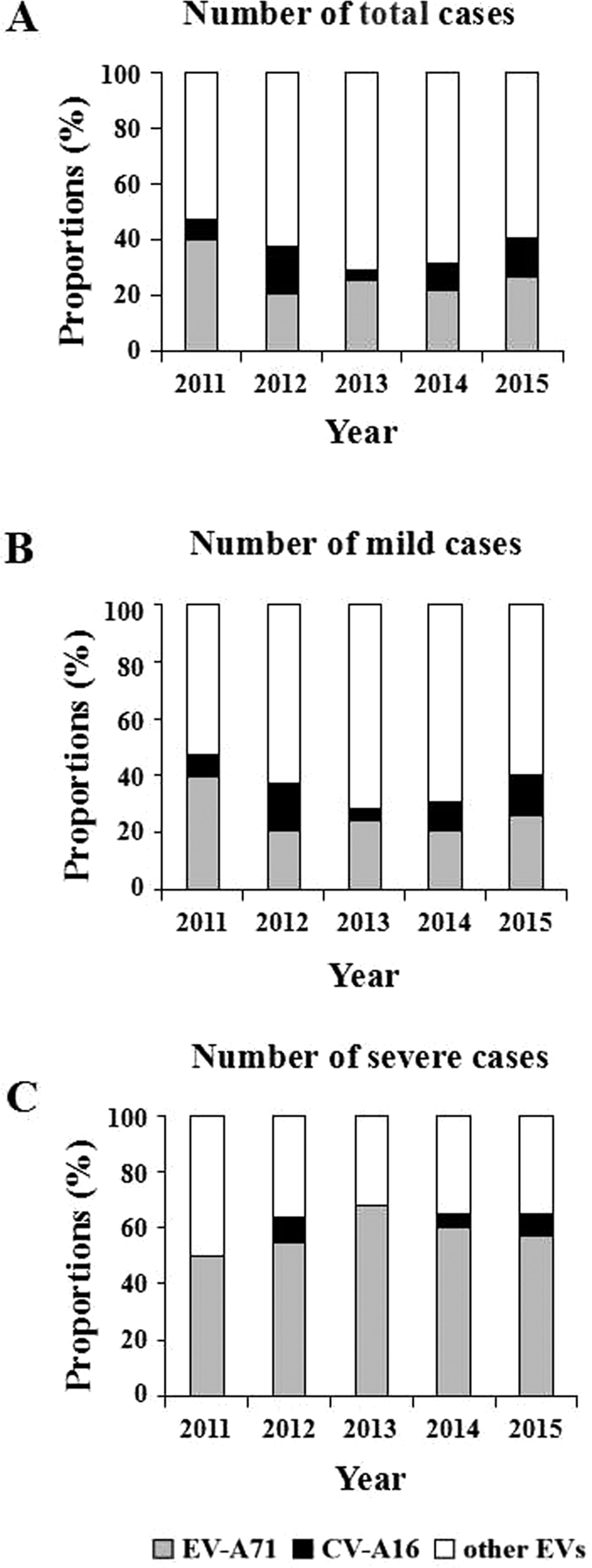
Proportions of enterovirus serotype in HFMD confirmed cases in Chongqing of China, 2011–2015. (**A**) Based on total cases. (**B**) Based on mild cases. (**C**) Based on severe cases. EV-A71 = enterovirus A71. CV-A16 = coxsackievirus A16. Other EVs = other enterovirus universal.

Of 21,615 confirmed HFMD cases, the positive rate of EV-A71 and CV-A16 was 25.13% (5431/21615) and 10.92% (2362/21615), respectively, whereas, other EVs dominated with 63.95% (13822/21615). The data for each year from 2011 to 2015 showed a similar pattern: the infection ratios of EV-A71 were higher than those of CV-A16. “Other EVs” accounted for more than 50% infection ratio, as follows: 52.67% (2011), 62.81% (2012), 70.85% (2013), 68.57% (2014), 59.39% (2015) correspondingly (Fig. 4A). In mild cases, other EVs were mainly pathogens (Fig. 4B), but in severe cases, EV-A71 was the main pathogen, accounting for 50.00% (2011), 54.55% (2012), 67.74% (2013), 60.36% (2014), 56.41% (2015) respectively (Fig. 4C).

**Figure 5:**
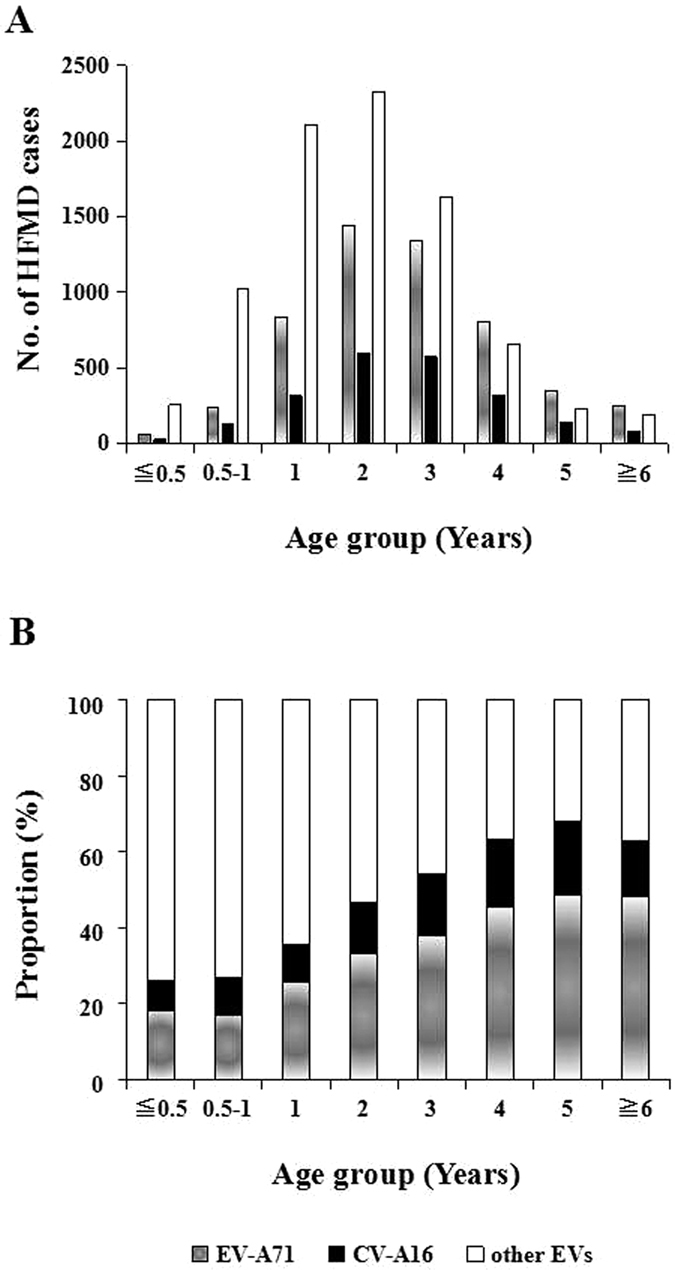
Enterovirus serotype distribution by age group in HFMD confirmed cases in Chongqing of China, 2011–2015. (**A**) Based on number of cases. (**B**) Based on proportion of serotypes.

**Figure 6:**
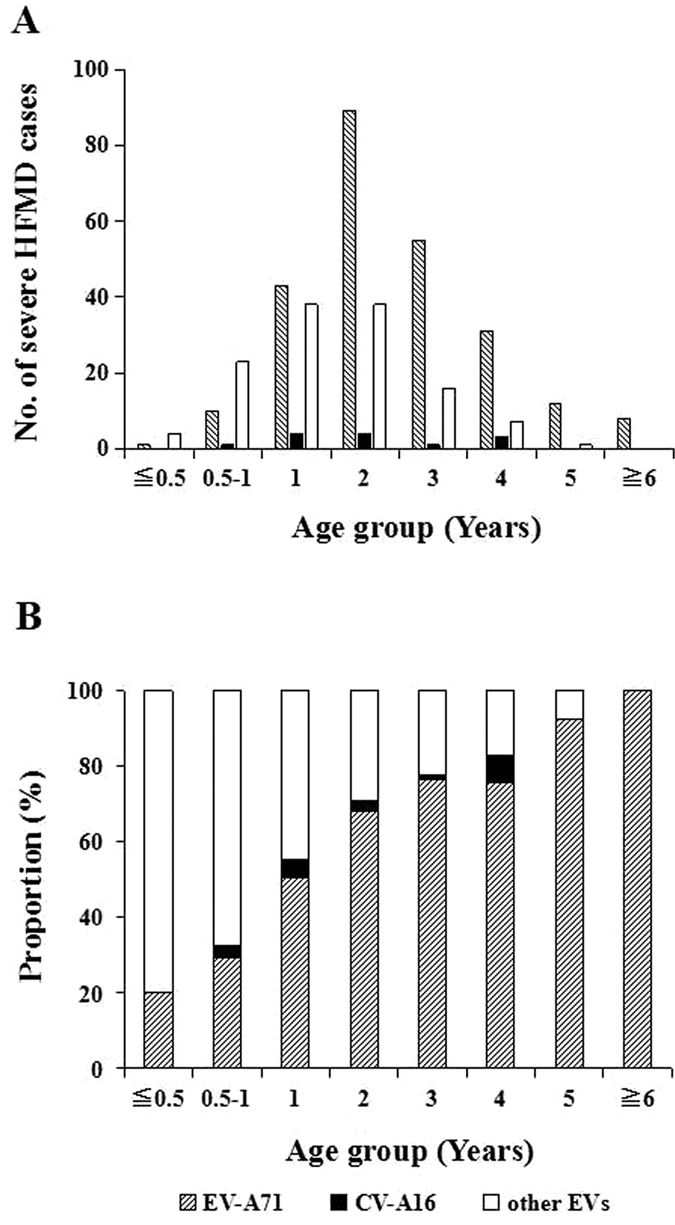
Enterovirus serotype distribution by age group in HFMD severe cases in Chongqing of China, 2011–2015. (**A**) Based on number of cases. (**B**) Based on proportion of serotypes.

All the EVs subtypes were age-specific, mainly infecting children aged 0.5–4 years. In general, EV-A71 was the main pathogen in children aged 4 years and above, which accounted for more than CV-A16 in each age group, whereas, other EVs were the main pathogen in children under 4 years (Fig. 5A). It became obvious that the proportion of EV-A71 and CV-A16 increased with age, but conversely the proportion of “other EVs” significantly decreased with age. EV-A71 increased from 18.08% to 48.40%, CV-A16 increased from 7.87% to 19.47%, “other EVs” decreased from 74.05% to 32.13% (Fig. 5B). In severe cases, EV-A71 was the main pathogen in children aged 1 year and above, CV-A16 accounted for a few in each age group, “other EVs” was only dominant in infants under 1 year (Fig. 6A). The trend of EV-A71 increased with age sharply, and increased to 100% in people aged more than 6 years. It is noteworthy that in children under 3 years old, “other EVs” has the highest percentage, which indicated other EVs were threating for severe HFMD in young children (Fig. 6B).

**Figure 7:**
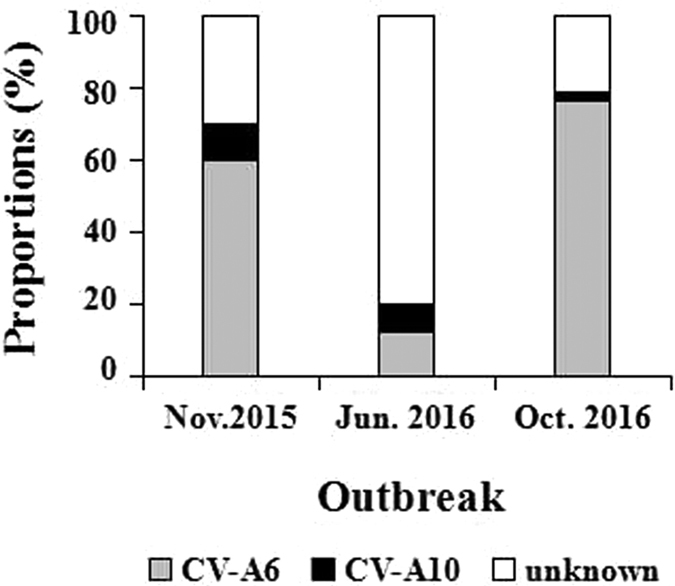
CV-A6 and CV-A10 detection in non-EV-A71 non-CV-A16 confirmed cases in three different outbreaks of November 2015, June 2016 and October 2016 in Chongqing of China.

In 292 non-EV-A71/CV-A16 laboratory confirmed HFMD cases, 182 cases were detected CV-A6 positive and 14 cases were detected CV-A10 positive without co-infection. 60% (30/50) were detected CV-A6 positive and 10% (5/50) were detected CV-A10 positive in the outbreak of November 2015, 12% (6/50) were detected CV-A6 positive and 8% (4/50) were detected CV-A10 positive in the outbreak of June 2016. 76.04% (146/192) were detected CV-A6 positive and 2.60% (5/192) were detected CV-A10 positive in the outbreak of October 2016 (Fig. 7). These results indicate that CV-A6 is highly prevalent in Chongqing with apparently season-varying, while CV-A10 occasionally cause HFMD with low incidence all the time.

## Discussion

HFMD is reported to have frequent outbreaks worldwide, especially in the Asian-Pacific region subsequent to the first reported outbreak in New Zealand in 1957. It was ranked as one of the infectious diseases with the highest incidence in China for five consecutive years, and was incorporated into the C class infectious diseases management in 2008. Our large-scale analysis of HFMD cases in Chongqing showed a bigger outbreak peak in Spring and a smaller one in Autumn without virus subtype-specific during 2011–2015. The main infectious age was in the 0.5–4 years and male children had a greater infections proportion. The EVs subtype distribution by age group was similar in both mild and severe HFMD cases. Their incidences increased with age until 2 years and then started to decrease. Children aged more than 6 years were seldom attacked by this disease, which was consistent with reports from other areas of China^22^,^23^,^24^,^25^,^26^,^27^. However, our results show that the HFMD population with the highest morbidity was the 2-year age group, which was different from the 1-year age group reported in the earlier epidemiology of HFMD^21^.

EV-A71 and CV-A16 have been widely considered to be the predominant pathogens and well-studied in HFMD cases in the past decades. Until 2015, the EV-A71 inactivated vaccine successfully entered the market in China^5^. In our EVs subtype analysis, other EVs infection accounted for 63.94% in the total HFMD cases while EV-A71 infection accounted for only 25.13% and CV-A16 infection was only 10.92%, but in severe cases, EV-A71 infection accounted for 59.64%. These results strongly emphasize that EV-A71 and CV-A16 are not the predominant pathogens in Chongqing, but nevertheless, a sharp vigilance is necessary for EV-A71 infection control. Results of EV-A71 show that vaccination would be very helpful in the prevention of severe cases, but a bivalent or polyvalent vaccine would be desirable, in light of the co-circulation of non-EV-A71/CV-A16 EVs, that are responsible for the highest proportion of HFMD morbidity and severe cases in patients within 1 year of age.

As demonstrated, both in our study and other reports^28^,^29^,^30^, the EV-A71 infection accounted for a large proportion in severe cases. It was taken as an independent risk factor in the severity development at all times. EVs subtype distribution by age group in this study illustrated that the proportion of EV-A71 infection was positively correlated with age, whereas, the proportion of “other EVs” infections was negatively correlated with age. In addition, the proportion of EV-A71 infection reached a maximum proportion of 48.4% in the 5-year age group in mild cases, whereas it increased more sharply in severe cases, reaching a proportion of 92.3% in the 5-year age group and completely dominating in the 6-year age group. Both in mild and severe cases, most HFMD children under 1 year were “other EVs” infected, thus demonstrating that morbidity in different causative respective infections, had different correlations with age. When evaluating the severity of HFMD, EVs subtype and age should be taken into consideration together as risk factors; neither could be considered independent risk factors if they are separated.

In recent years, numerous epidemiology studies in different areas proved that pathogens in circulation kept drifting^8^,^9^,^10^,^11^,^12^,^13^,^14^,^15^, which was likely the main reason why “other EVs” infections accounted for 63.94% in our study. Among “other EVs”, CV-A6 and CV-A10 emerged as the most prominent new pathogens. To investigate their epidemic proportions in Chongqing, we randomly collected 292 “other EVs” positive only samples, in three different outbreaks, for simultaneous detection of CV-A6 and CV-A10. 62.33% (182/292) were detected CV-A6 positive and 4.79% (14/292) were detected CV-A10 positive without co-infection, which demonstrated that most non-EV-A71/CV-A16 laboratory confirmed HFMD cases could be accurately classified with simultaneously detecting CV-A6 and CV-A10. Noteworthy is the fact that CV-A6 positive cases accounted up for 60% and 76.04% of non-EV-A71/CV-A16 HFMD confirmed cases in the outbreak of November 2015 and October 2016, respectively, but only 12% in the outbreak of June 2016. CV-A10 positive cases accounted for only 10%, 8% and 2.60% of non-EV-A71/CV-A16 HFMD confirmed cases in the outbreak of November 2015, June 2016 and October 2016. These results suggested that CV-A6 and CV-A10 were prevalent in this area. CV-A6 would be more likely to experience an outbreak while CV-A10 would remain relatively stable and occur sporadically.

CV-A6 infection should be considered in the differential diagnosis of an acute exacerbation of atopic dermatitis (AD)^31^ and herpes zoster^32^, particularly in children, and specific warnings should be given to patients with CV-A6 infection because desquamation, onychomadesis or nail abnormalities can occur between one week to two months^16^,^17^,^18^,^19^,^20^. CV-A10 infection has most commonly been associated with more mild disease^33^ while infections with many other EVs serotypes may be severe and even fatal. Specific medications^34^,^35^,^36^,^37^ could be administered and actions taken on corresponding patients with risk factors^38^,^39^,^40^,^41^,^42^ while most mildly infected patients only need the expected conventional treatment. In our study, CV-A6/CV-A10 positive rates reached up to 67.12% in non-EV-A71/CV-A16 HFMD laboratory confirmed cases. Therefore, CV-A6/CV-A10 detection should be incorporated into the conventional etiologic test, which would be necessary in the different diagnosis and severity evaluation of HFMD.

Taking into account all these factors and findings, our study clearly demonstrates that causative EVs of HFMD is pluralistic. Simultaneously detecting CV-A6 and CV-A10 in a clinical laboratory in combination with EV-A71 and CV-A16, could greatly enhance EVs subtype identification.

There are some limitations to the study. For example, most “other EVs” positive only samples between 2011 and 2015 could not be collected. CV-A6 and CV-A10 analysis might show bias because of fewer samples and the short time duration, even though these results were consistent with clinical reports from other regions of China^8^,^9^,^10^,^11^,^12^,^13^,^14^,^15^. However, (1) the long-term virological surveillances of HFMD should be on going, and (2) a much broader range of EVs subtype diagnostic methods and kits are necessary in any further outbreaks of HFMD.

## Additional Information

**How to cite this article:** Tao, J. *et al*. Epidemiology of 45,616 suspect cases of Hand, Foot and Mouth Disease in Chongqing, China, 2011-2015. *Sci. Rep.* **7**, 45630; doi: 10.1038/srep45630 (2017).

**Publisher's note:** Springer Nature remains neutral with regard to jurisdictional claims in published maps and institutional affiliations.

## Figures and Tables

**Table 1:** Month, age, gender and serotype distribution of HFMD confirmed cases between 2011 and 2015 in Chongqing of China.

	HFMD confirmed cases, n (%)				
	2011	2012	2013	2014	2015
Month					
Jan	15 (0.66)	28 (0.64)	131 (3.68)	136 (2.15)	242 (4.76)
Feb	9 (0.40)	28 (0.64)	47 (1.32)	88 (1.39)	88 (1.73)
Mar	38 (1.67)	169 (3.87)	170 (4.77)	268 (4.24)	138 (2.71)
Apr	230 (10.10)	328 (7.50)	375 (10.52)	791 (12.52)	526 (10.35)
May	550 (24.15)	732 (16.74)	533 (14.96)	790 (12.50)	900 (17.71)
Jun	464 (20.38)	648 (14.82)	492 (13.80)	865 (13.69)	1408 (27.70)
Jul	223 (9.79)	445 (10.18)	336 (9.43)	435 (6.88)	611 (12.02)
Aug	71 (3.12)	176 (4.03)	102 (2.86)	151 (2.39)	116 (2.28)
Sep	45 (2.00)	184 (4.21)	205 (5.750	538 (8.51)	135 (2.66)
Oct	228 (10.01)	605 (13.84)	431 (12.09)	1040 (16.46)	348 (6.85)
Nov	282 (12.38)	574 (13.13)	399 (11.20)	808 (12.79)	395 (7.77)
Dec	122 (5.36)	455 (10.41)	343 (9.62)	409 (6.47)	176 (3.46)
Age group					
<0.5y	25 (1.10)	44 (1.01)	51 (1.43)	92 (1.46)	52 (1.02)
0.5–1y	98 (4.30)	386 (8.83)	430 (12.07)	620 (9.81)	434 (8.54)
1y	449 (19.72)	754 (17.25)	833 (23.37)	1016 (16.08)	1074 (21.13)
2y	634 (27.84)	1158 (26.49)	862 (24.19)	2274 (35.99)	1461 (28.74)
3y	618 (27.14)	1088 (24.89)	762 (21.38)	1219 (19.29)	1190 (23.41)
4y	303 (13.31)	541 (12.37)	377 (10.58)	649 (10.27)	508 (9.99)
5y	81 (3.56)	241 (5.51)	141 (3.96)	250 (3.96)	223 (4.39)
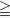 6y	69 (3.03)	160 (3.66)	108 (3.03)	199 (3.15)	141 (2.77)
Sex					
Male	1410 (61.92)	2689 (61.51)	2169 (60.86)	3761 (59.52)	3061 (60.22)
Female	867 (38.08)	1683 (38.49)	1395 (39.14)	2558 (40.48)	2022 (39.78)
Enterovirus serotype					
EV-A71	901 (39.57)	903 (20.65)	895 (25.11)	1376 (21.78)	1356 (26.68)
CV-A16	177 (7.77)	723 (16.54)	144 (4.04)	610 (9.65)	708 (13.93)
Other EVs	1199 (52.66)	2746 (62.81)	2525 (70.85)	4333 (68.57)	3019 (59.39)
Total	2277 (100.00)	4372 (100.00)	3564 (100.00)	6319 (100.00)	5083 (100.00)

HFMD = hand, foot and mouth disease. EV-A71 = enterovirus A71. CV-A16 = coxsackievirus A16. Other EVs = other enteroviruses universal.
